# Real-World Safety and Herpes Zoster Outcomes After Recombinant Zoster Vaccination in Inflammatory Bowel Disease

**DOI:** 10.3390/jcm15135310

**Published:** 2026-07-07

**Authors:** Gian Mario Morrone, Sara Sandri, Marta Vernero, Angelo Armandi, Gian Paolo Caviglia, Davide Giuseppe Ribaldone

**Affiliations:** 1 Department of Medical Sciences, University of Turin, 10126 Turin, Italy; marta.vernero@unito.it (M.V.); angelo.armandi@unito.it (A.A.); gianpaolo.caviglia@unito.it (G.P.C.); davidegiuseppe.ribaldone@unito.it (D.G.R.); 2 University of Turin, 10126 Turin, Italy; sara.sandri585@edu.unito.it

**Keywords:** inflammatory bowel disease, herpes zoster, Shingrix, vaccination, advanced therapy, safety

## Abstract

**Background/Objectives:** Patients with inflammatory bowel disease (IBD) are at an increased risk of herpes zoster (HZ), particularly during immunosuppressive treatment. To assess the safety of the recombinant zoster vaccine (RZV, Shingrix^®^) and describe HZ occurrence in a real-world IBD cohort, including patients receiving advanced therapies. **Methods:** This prospective, single-centre observational study included 114 adults with IBD who were offered RZV; 69 received at least one dose, and 45 declined or postponed vaccination. Follow-up began at the vaccine proposal. For time-to-event analyses, vaccination was modelled as a time-varying exposure beginning 14 days after a documented second dose. IBD clinical relapse was defined by worsening disease-activity indices and/or treatment escalation. **Results:** During a median follow-up of 17 months (IQR 9–29), 11 patient-reported HZ episodes occurred: 8/69 (11.6%) among patients who received vaccination and 3/45 (6.7%) among those who did not. The time-dependent association between complete vaccination and HZ was not statistically significant (HR 2.51; 95% CI 0.66–9.51; *p* = 0.18), and adjustment for advanced therapy did not materially change the estimate. Prior HZ was associated with a higher risk of a subsequent reported episode (adjusted HR 6.26; 95% CI 1.80–21.76; *p* = 0.004). IBD clinical relapse occurred in 4/69 (5.8%) vaccinated and 2/45 (4.4%) unvaccinated patients (*p* = 1.00). Among 72 patients receiving advanced therapy, HZ occurred in 4/42 vaccinated and 2/30 unvaccinated patients (*p* = 1.00), while IBD relapse occurred in 3/42 and 1/30, respectively (*p* = 0.64). No serious vaccine-related adverse events or significant pre-/post-vaccination changes in disease activity were observed. **Conclusions:** RZV showed a favourable short-term safety profile in patients with IBD, including those receiving advanced therapies. The small number of self-reported HZ events and the non-randomised design preclude conclusions regarding vaccine effectiveness.

## 1. Introduction

Inflammatory bowel diseases (IBD), encompassing ulcerative colitis (UC) and Crohn’s disease (CD), represent an increasingly significant clinical challenge, with a global prevalence estimated at approximately 229.7 cases per 100,000 individuals and a mean incidence of 9.7 new cases per 100,000 person-years [1,2]. Beyond gastrointestinal involvement, patients with IBD exhibit an intrinsic susceptibility to opportunistic infections, due to complex dysregulation of mucosal barrier function and immune responses. This impairment results in suboptimal protection against pathogens and reduced control of latent infections, conferring a 1.2- to 2-fold higher risk of varicella–zoster virus (VZV) reactivation compared with the general population [3,4]. In this setting, herpes zoster (HZ) not only occurs more frequently but also tends to present with more severe clinical phenotypes, including multidermatomal and disseminated forms, as well as ocular and neurological complications. Moreover, the occurrence of HZ and postherpetic neuralgia (PHN) may necessitate the discontinuation of immunomodulatory therapies, thereby compromising disease control.

The risk of infection is further increased by the use of immunosuppressive agents. While the impact of corticosteroids and thiopurines is well established, owing to their inhibitory effects on lymphocyte function, the advent of Janus kinase (JAK) inhibitors has introduced an additional concern. Meta-analyses indicate that agents such as tofacitinib and upadacitinib are associated with a 4- to 6-fold increased risk of HZ compared with the general population [5,6,7]. In contrast, anti-integrin agents such as vedolizumab and anti–interleukin-12/23 therapies such as ustekinumab have not been consistently associated with an increased risk of herpes zoster in available long-term safety data [8].

In this context, the adjuvanted recombinant zoster vaccine (RZV; Shingrix^®^) has reshaped preventive strategies. Unlike the previously available live attenuated zoster vaccine (LZV), RZV is a subunit vaccine composed of the VZV glycoprotein E (gE) and the AS01B adjuvant system. The latter contains monophosphoryl lipid A (MPL) and the saponin QS-21, which act synergistically to enhance both innate and adaptive immune responses, promoting robust activation of VZV-specific CD4+ T cells [9]. The high clinical efficacy of RZV has been demonstrated in the phase 3 ZOE-50 and ZOE-70 trials. In ZOE-50, conducted in more than 15,000 adults, the vaccine showed an efficacy of 97.2% in preventing HZ, whereas ZOE-70 and pooled analyses confirmed a protective efficacy of 91.3% among individuals aged 70 years or older. In addition, the vaccine conferred near-complete protection (close to 100%) against PHN in adults older than 50 years [10,11].

Despite these advances, important knowledge gaps remain in the management of patients with IBD. Although recent prospective studies indicate that RZV is both immunogenic and safe in patients receiving biologic therapies, including anti–tumour necrosis factor agents and vedolizumab [12], without triggering disease flares [13,14], much of the available evidence derives from post hoc analyses or observational studies. Notably, the original pivotal ZOE trials systematically excluded immunocompromised individuals, leaving uncertainty regarding the impact of newer therapies—such as JAK inhibitors—on vaccine-specific immune responses. Furthermore, long-term data on the durability of protection in individuals vaccinated before 50 years of age remain limited, and robust clinical data in paediatric populations are lacking.

Current international guidelines (including those from the American College of Gastroenterology and the European Crohn’s and Colitis Organisation) emphasise the importance of early vaccination planning, ideally before the initiation of immunosuppressive therapy. Vaccination is strongly recommended for all patients with IBD aged 50 years or older, as well as for younger adults (≥18 years) who are candidates for or are receiving immunomodulatory treatments [15,16]. Addressing the remaining evidence gaps—particularly with respect to optimal timing in relation to biologic therapy—remains a priority to refine immunisation strategies in this complex patient population.

## 2. Materials and Methods

### 2.1. Study Design and Population

This prospective, single-centre observational study was conducted at the Inflammatory Bowel Disease (IBD) outpatient clinic of the “San Giovanni Antica Sede” Hospital, Turin, Italy.

Adult patients (≥18 years) with a confirmed diagnosis of Crohn’s disease (CD), ulcerative colitis (UC), or IBD-unclassified (IBD-U) were eligible. Patients with incomplete baseline or follow-up data were excluded. All participants were followed within routine clinical practice.

### 2.2. Vaccination Procedure

The recombinant adjuvanted zoster vaccine (RZV, Shingrix^®^) was offered according to standard clinical practice. Vaccination was recommended for patients eligible for or receiving biologic agents or small-molecule therapies and for patients with a prior history of HZ, provided that at least 6 months had elapsed since the acute episode.

During outpatient visits, patients were informed about the vaccine indications, expected benefits, and potential adverse events, and the recommendation was recorded in the medical notes. RZV was administered intramuscularly as a two-dose series, with the second dose scheduled 2–6 months after the first.

### 2.3. Data Collection

The following variables were collected: demographic characteristics, smoking status, IBD subtype and duration, baseline disease activity, prior HZ, IBD therapy at the start of follow-up, vaccination dates, age at vaccination, local and systemic adverse events, reported HZ episodes, and IBD clinical relapse.

HZ outcomes were collected by a structured telephone self-report, including the reported date, severity, and complications. Independent dermatologic assessment or virologic confirmation was not required for study inclusion; therefore, these outcomes are referred to as patient-reported HZ episodes throughout the manuscript.

Disease activity was assessed before and after vaccination using validated indices: the Harvey–Bradshaw Index (HBI) for CD and the partial Mayo score for UC or IBD-U. Clinical remission was defined as HBI ≤ 4 and partial Mayo score ≤ 2.

IBD clinical relapse was defined as worsening of the HBI or partial Mayo score and/or the need for treatment modification, including dose escalation, a switch of therapy, or the initiation of corticosteroids.

### 2.4. Outcomes

The primary outcome was the occurrence of a patient-reported HZ episode during follow-up. Vaccine effectiveness was considered exploratory because the study was not powered for a between-group effectiveness estimate.

### 2.5. Secondary Outcomes

Secondary outcomes were vaccine safety and tolerability, assessed through the structured recording of local and systemic adverse events and the need for medical attention; the impact of HZ occurrence on the continuity of IBD treatment, including interruption, delay, or modification; HZ-related complications, particularly post-herpetic neuralgia; and changes in IBD activity, including IBD clinical relapse and paired pre-/post-vaccination HBI or partial Mayo scores.

### 2.6. Statistical Analysis

Continuous variables are reported as the median and interquartile range (IQR), and categorical variables as number and percentage.

Independent continuous variables were compared using the Mann–Whitney U test, paired pre-/post-vaccination values using the Wilcoxon signed-rank test, and categorical variables using the chi-square test or Fisher’s exact test when expected cell counts were small. Advanced therapy was defined as treatment with a biologic agent or small molecule; azathioprine was classified as a conventional immunomodulator.

Follow-up began on the date of the vaccine proposal for every patient, irrespective of subsequent uptake, and ended at the first reported HZ episode, the last available clinical contact, or database lock. This common time origin avoided assigning a later baseline to vaccinated patients.

To address changes in vaccination status and potential immortal time bias, vaccination was modelled as a time-varying exposure. Patients contributed unvaccinated person-time from the vaccine-proposal date until 14 days after the documented second dose and vaccinated person-time thereafter. Patients without a documented second-dose date remained in the unvaccinated risk set for the time-dependent analysis. Kaplan–Meier curves by eventual vaccination status were retained only as an unadjusted descriptive display and were not used as the primary effectiveness analysis.

Cox proportional-hazards regression was used to estimate associations with reported HZ. Because only 11 events occurred, no stepwise or data-driven variable-selection procedure was used. Two separate parsimonious adjusted models were specified a priori: time-varying vaccination plus prior HZ, and time-varying vaccination plus advanced therapy. Each model contained two covariates (5.5 events per variable). Exploratory univariate analyses included demographic and disease-related variables; the HBI was evaluated only in patients with CD, and the partial Mayo score only in patients with UC/IBD-U.

Descriptive analyses were performed using MedCalc Statistical Software (version 23.1.2; MedCalc Software Ltd., Ostend, Belgium). The reviewer-requested time-varying Cox analyses were performed in Python 3.11 using statsmodels version 0.14.6. Two-sided *p*-values < 0.05 were considered statistically significant; given the limited number of events, adjusted estimates are interpreted as exploratory.

### 2.7. Use of Generative Artificial Intelligence

During the revision of the manuscript, OpenAI ChatGPT (GPT-5.5 Pro) was used to assist with English-language editing, the organisation of responses to peer reviewers, and the formulation and checking of statistical code for the reviewer-requested sensitivity analyses. The tool was not used to define eligibility, adjudicate outcomes, generate or alter source data, or make final scientific decisions. All text and numerical outputs were reviewed and verified by the authors against the source database, and the authors take full responsibility for the content of the manuscript.

## 3. Results

Between October 2021 and January 2026, RZV vaccination was recommended to 126 patients with IBD. Of these, 114 were successfully contacted and included in the analysis: 69 (60.5%) received at least one vaccine dose, while 45 (39.5%) declined or postponed vaccination.

Among the 69 patients who received at least one dose, 36 (52.2%) were male and 33 (47.8%) were female; 44 (63.8%) had CD, 21 (30.4%) had UC, and 4 (5.8%) had IBD-U.

A second-dose administration date was documented for 65/69 patients (94.2%). Second-dose safety information was available for 66 patients, including one patient for whom the administration date had not been recorded in the study database.

Baseline demographic and clinical characteristics were broadly comparable between groups. Advanced-therapy use was similar (42/69 [60.9%] vaccinated vs. 30/45 [66.7%] unvaccinated; *p* = 0.56), whereas follow-up was longer in the unvaccinated group (median 23 vs. 16 months; *p* = 0.02) (Table 1).

IBD therapy data at the start of follow-up were available for all 114 patients, including all 45 unvaccinated patients. Of the unvaccinated group, 30/45 (66.7%) were receiving advanced therapy, and 15/45 (33.3%) were not receiving advanced or conventional immunomodulatory therapy (Table 2).

### 3.1. Reported Herpes Zoster During Follow-Up

During a median follow-up of 17 months (IQR 9–29), 11 patient-reported HZ episodes occurred: 8/69 (11.6%) among patients who received vaccination and 3/45 (6.7%) among those who did not (Fisher’s exact *p* = 0.52). Seven of the 11 patients (63.6%) reported a prior HZ episode. In the descriptive Kaplan–Meier analysis by eventual vaccination status, the log-rank *p*-value was 0.22. In the primary time-dependent analysis, complete vaccination was not significantly associated with HZ (HR 2.51; 95% CI 0.66–9.51; *p* = 0.18).

No reported episode was associated with post-herpetic neuralgia or another recorded HZ-related complication.

**Figure 1:**
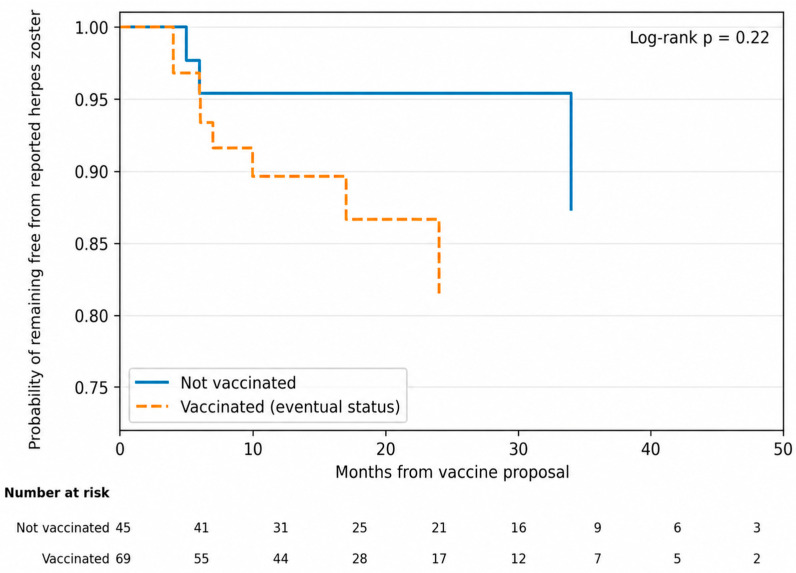
Descriptive Kaplan–Meier curves for remaining free from patient-reported herpes zoster, grouped by eventual vaccination status. Vaccination was modelled as a time-varying exposure in the Cox analyses.

Figure 1 presents the unadjusted Kaplan–Meier curves by eventual vaccination status. Because this display assigns patients according to their eventual group, it is descriptive; the time-varying Cox analysis is the inferential analysis used to address immortal time bias.

### 3.2. Safety

Adverse events were collected through structured telephone follow-up after each dose.

Prespecified reactogenicity outcomes included fever (>37.5 °C), injection-site reactions, other systemic symptoms (including fatigue, headache, myalgia/arthralgia, chills, or gastrointestinal symptoms), lymphadenopathy, and more intense reactions causing flu-like symptoms for ≥2–3 days or temporary limitation of daily activities.

After dose 1 (*n* = 69), fever was reported by 21 patients (30.4%), injection-site reactions by 18 (26.1%), other systemic symptoms by 16 (23.2%), lymphadenopathy by 3 (4.3%), and more intense reactions by 5 (7.2%).

Second-dose safety data were available for 66 patients: fever was reported by 22 (33.3%), injection-site reactions by 20 (30.3%), other systemic symptoms by 15 (22.7%), lymphadenopathy by 2 (3.0%), and more intense reactions by 5 (7.6%).

**Figure 2:**
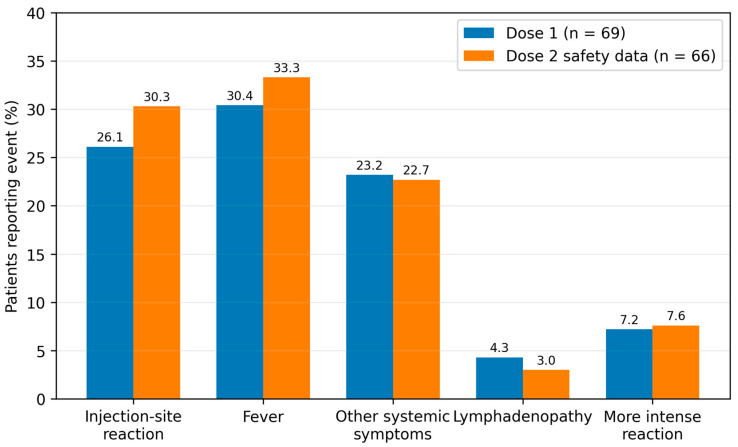
Local and systemic reactogenicity after the first and second RZV doses. Percentages are based on available safety data for each dose.

Reactogenicity profiles were similar after the two doses (Figure 2).

Most adverse events were mild to moderate and self-limiting; no serious vaccine-related adverse event was recorded.

### 3.3. IBD Clinical Relapse

During follow-up, six IBD clinical relapses were recorded: 4/69 (5.8%) in vaccinated patients and 2/45 (4.4%) in unvaccinated patients, with no statistically significant difference (Fisher’s exact *p* = 1.00).

### 3.4. Predictors of Reported Herpes Zoster

In univariate Cox analyses, prior HZ was associated with a higher risk of another reported episode (HR 6.46; 95% CI 1.87–22.35; *p* = 0.003). Higher baseline HBI was also associated with risk in the CD-only analysis (HR 2.06; 95% CI 1.01–4.21; *p* = 0.046). Advanced therapy was not associated with HZ (HR 0.66; 95% CI 0.20–2.17; *p* = 0.50).

Because HBI and partial Mayo scores are disease-specific and only 11 events occurred, disease-activity indices were not entered into the overall adjusted models. In the model including time-varying vaccination and prior HZ, vaccination was not significant (HR 2.29; 95% CI 0.61–8.65; *p* = 0.22), whereas prior HZ remained associated with risk (HR 6.26; 95% CI 1.80–21.76; *p* = 0.004). In the model including time-varying vaccination and advanced therapy, neither vaccination (HR 2.51; 95% CI 0.66–9.54; *p* = 0.18) nor advanced therapy (HR 0.66; 95% CI 0.20–2.17; *p* = 0.49) was significant.

The two parsimonious adjusted models are shown in Table 3. Their estimates are exploratory because each model was based on 11 events and two covariates (5.5 events per variable).

### 3.5. Advanced-Therapy Subgroup Analysis

Seventy-two patients were receiving advanced therapy at the start of follow-up: 42 vaccinated and 30 unvaccinated. HZ was reported by 4/42 (9.5%) vaccinated and 2/30 (6.7%) unvaccinated patients (Fisher’s exact *p* = 1.00); the time-dependent vaccination HR within this subgroup was 1.90 (95% CI 0.35–10.41; *p* = 0.46). IBD clinical relapse occurred in 3/42 (7.1%) and 1/30 (3.3%), respectively (*p* = 0.64). Among vaccinated patients receiving advanced therapy, paired disease-activity data showed no significant pre-/post-vaccination change in HBI for CD (*n* = 27; *p* = 0.76) or partial Mayo score for UC/IBD-U (*n* = 11; *p* = 0.54).

### 3.6. Disease Activity Before and After Vaccination

Paired pre-/post-vaccination disease-activity data were available for 38 vaccinated patients with CD and 25 with UC/IBD-U.

The HBI remained stable at a median of 2 (IQR 2–3) before and after vaccination (*p* = 0.50). The partial Mayo score also did not differ significantly (median 3 [IQR 2–3] before vs. 3 [IQR 2–4] after vaccination; *p* = 0.19).

The complete baseline characteristics, treatment distribution and time-dependent Cox models are provided in the Supplementary Materials (Tables S1–S3).

## 4. Discussion

This prospective real-world study evaluated RZV safety and described patient-reported HZ outcomes in patients with IBD, with particular attention to those receiving biologic agents or small molecules. Although the study was not designed or powered to establish vaccine effectiveness, it provides clinically relevant information regarding vaccine tolerability in routine practice.

RZV is a non-live vaccine and is therefore suitable for patients receiving immunomodulatory treatment. The clinically relevant question in this setting is whether vaccination is tolerated without increasing IBD activity while patients remain on advanced therapy.

Of 114 included patients, 69 (60.5%) received at least one RZV dose. This uptake remains suboptimal and supports structured counselling, recall systems, shared scheduling, and simplified access. The observed uptake is consistent with broader population-based data showing low shingles vaccination coverage among eligible adults [17,18].

Measured baseline characteristics and the overall prevalence of advanced therapy were similar between vaccinated and unvaccinated patients. However, unvaccinated patients had longer follow-up, and treatment allocation was not random. These features reinforce the need for time-to-event methods and leave scope for residual confounding and selection bias despite statistical adjustment.

Prior HZ was the strongest observed predictor of another reported episode. Higher HBI was associated with risk in the CD-only univariate analysis, but the low event count and disease-specific nature of HBI precluded a stable overall multivariable assessment. The adjusted time-dependent models therefore focused on the exposure of interest, prior HZ, and advanced therapy using separate parsimonious models.

No statistically significant protective association was observed between complete vaccination and patient-reported HZ. The point estimates were imprecise and the confidence intervals were wide, reflecting only 11 events. Accordingly, the higher crude number of events in the vaccinated group should not be interpreted as evidence of harm, and the absence of a significant difference should not be interpreted as evidence of vaccine ineffectiveness. This cohort cannot provide a reliable effectiveness estimate.

Although our cohort was underpowered to demonstrate a significant reduction in herpes zoster incidence, previous real-world evidence in patients with IBD has suggested that RZV may be associated with a reduced risk of herpes zoster [19]. Differences in sample size, outcome ascertainment, confounding control, prior-HZ prevalence, treatment exposure, and follow-up duration limit direct comparison with the present study. Evidence from other immunocompromised populations also supports clinically relevant protection, although estimates may be lower than in immunocompetent registration trials [20,21].

The principal finding of the present study concerns safety. Reactogenicity was generally mild to moderate and self-limiting, no serious vaccine-related event was recorded, and IBD clinical relapse rates were similar between vaccinated and unvaccinated patients. Paired HBI and partial Mayo scores did not show significant worsening after vaccination.

These safety findings are consistent with prior IBD studies reporting a low flare rate after RZV and no increase in disease exacerbation [14,19]. Our HZ outcome ascertainment was less rigorous because episodes were self-reported and not systematically clinically or virologically confirmed; misclassification is therefore possible and may partly explain the observed event frequency.

The advanced-therapy subgroup is of particular clinical relevance. Among 72 patients receiving a biologic or small molecule, vaccination was not associated with a statistically significant increase in HZ or IBD clinical relapse, and disease-activity indices remained stable in vaccinated patients with paired data. These results support short-term safety during advanced therapy, but the subgroup contained only six HZ events and four relapses, so comparative conclusions remain uncertain.

Current guidance emphasises early vaccination planning, ideally before immunosuppression when feasible, while also recommending RZV for adults who are receiving or will receive immunomodulatory treatment [15,22]. In practice, many patients are already receiving advanced therapy when vaccination is considered; the present safety data are therefore relevant to routine care.

Vaccination decisions should be individualised according to age, type and intensity of immunosuppression, disease activity, and prior HZ. Clear counselling and coordinated vaccination pathways remain important to improve uptake.

This study has several limitations. It was conducted at a single centre and included a modest, non-randomised cohort, creating potential selection bias and residual confounding. Only 11 HZ events occurred, limiting statistical power and yielding fewer events per model parameter than is desirable for stable multivariable estimation. Follow-up differed between groups, although a common time origin and time-varying vaccination exposure were used to reduce immortal time bias. HZ episodes were self-reported and were not systematically confirmed by clinical examination or virologic testing, allowing outcome misclassification. A second-dose date was not documented for four patients, vaccine immunogenicity was not measured, and treatment changes during follow-up were not modelled as time-varying exposures.

Despite these limitations, this study provides additional real-world evidence supporting the short-term safety and tolerability of RZV in patients with IBD, including those receiving advanced therapies. While the present cohort cannot establish vaccine effectiveness, the findings should reassure clinicians that vaccination does not appear to adversely affect disease activity and may be safely incorporated into routine IBD care.

## 5. Conclusions

This real-world cohort supports the short-term safety of RZV in patients with IBD, including those receiving biologic agents and small molecules. IBD clinical relapse and paired disease-activity indices did not indicate any worsening after vaccination. However, the low number of self-reported HZ events, the single-centre design, the non-randomised comparison, and the wide confidence intervals preclude conclusions about vaccine effectiveness.

Larger multicentre studies with longer follow-up, clinically or virologically confirmed outcomes, and robust control of time-varying treatment and vaccination exposure are needed to define effectiveness across therapeutic subgroups and the durability of protection in immunocompromised patients.

Improving vaccine uptake and documenting complete dosing remain practical priorities in IBD care.

## Figures and Tables

**Table 1:** Baseline characteristics and follow-up of vaccinated and unvaccinated patients.

Variable	Vaccinated (*n* = 69)	Not Vaccinated (*n* = 45)	*p*-Value
IBD duration, years	11 (3–20)	11 (3–18)	0.46
Age at follow-up start, years	47 (35–58)	45 (35–62)	0.78
Follow-up duration, months	16 (8–24)	23 (10–34)	0.02
Female sex	33 (47.8%)	18 (40.0%)	0.45
Male sex	36 (52.2%)	27 (60.0%)	—
Crohn’s disease	44 (63.8%)	32 (71.1%)	0.54
Ulcerative colitis	21 (30.4%)	13 (28.9%)	—
IBD-U	4 (5.8%)	0 (0%)	—
Prior herpes zoster	14 (20.3%)	9 (20.0%)	1.00
Advanced therapy	42 (60.9%)	30 (66.7%)	0.56
Baseline HBI in CD	2 (2–3)	2 (2–3)	0.43
Baseline partial Mayo in UC/IBD-U	3 (2–3)	2 (2–2)	0.09

Note: Values are median (IQR) or *n* (%). Advanced therapy includes biologic agents and small molecules; azathioprine is classified as a conventional immunomodulator. IBD-U, inflammatory bowel disease-unclassified; HBI, Harvey–Bradshaw Index; IQR, interquartile range.

**Table 2:** IBD therapy at the start of follow-up, by vaccination group (*n* = 114).

Therapy	Vaccinated	Not Vaccinated
Adalimumab	19 (27.5%)	11 (24.4%)
Azathioprine	1 (1.4%)	0 (0%)
Filgotinib	1 (1.4%)	0 (0%)
Infliximab	4 (5.8%)	9 (20.0%)
Risankizumab	2 (2.9%)	1 (2.2%)
Tofacitinib	1 (1.4%)	0 (0%)
Upadacitinib	3 (4.3%)	0 (0%)
Ustekinumab	7 (10.1%)	4 (8.9%)
Ustekinumab + vedolizumab	1 (1.4%)	0 (0%)
Vedolizumab	4 (5.8%)	5 (11.1%)
No advanced/conventional therapy	26 (37.7%)	15 (33.3%)
Any advanced therapy	42 (60.9%)	30 (66.7%)
Total	69 (100%)	45 (100%)

Note: Percentages use the column denominator. Advanced therapy includes biologic agents and small molecules. The “any advanced therapy” row is a summary and is not additive to the individual drug rows.

**Table 3:** Time-dependent Cox models for patient-reported herpes zoster.

Model and Variable	HR (95% CI)	*p*-Value
Model 1: Vaccination (time-varying)	2.29 (0.61–8.65)	0.22
Model 1: Prior herpes zoster	6.26 (1.80–21.76)	0.004
Model 2: Vaccination (time-varying)	2.51 (0.66–9.54)	0.18
Model 2: Advanced therapy	0.66 (0.20–2.17)	0.49

Note: Vaccination was modelled as a time-varying exposure beginning 14 days after a documented second dose. Advanced therapy includes biologic agents and small molecules. CI, confidence interval; HR, hazard ratio.

## Data Availability

The original contributions presented in this study are included in the article. Further inquiries can be directed to the corresponding author.

## Supplementary Materials

The following supporting information can be downloaded at: https://www.mdpi.com/article/10.3390/jcm15135310/s1. Table S1: Baseline characteristics and follow-up according to vaccination status; Table S2: IBD therapy at the start of follow-up according to vaccination status; Table S3: Time-dependent Cox models for patient-reported herpes zoster.

## Abbreviations

The following abbreviations are used in this manuscript:

ACG	American College of Gastroenterology
AS01	Adjuvant System 01
CD	Crohn’s Disease
CI	Confidence Interval
CMV	Cytomegalovirus
ECCO	European Crohn’s and Colitis Organisation
HBI	Harvey Bradshaw Index
HBV	Hepatitis B Virus
HCV	Hepatitis C Virus
HIV	Human Immunodeficiency Virus
HR	Hazard Ratio
IBD	Inflammatory Bowel Disease
IBD-U	Inflammatory Bowel Disease Unclassified
IQR	Interquartile Range
RZV	Recombinant Zoster Vaccine
UC	Ulcerative Colitis
VZV	Varicella-Zoster Virus
