# Supplementary material for: Real-World Safety and Herpes Zoster Outcomes After Recombinant Zoster Vaccination in Inflammatory Bowel Disease
Source: J Clin Med. 2026 Jul 7;15(13):5310. doi: 10.3390/jcm15135310 (PMC13362674; doi:10.3390/jcm15135310)
Supplement: Supplementary file 1 [file jcm-15-05310-s001.zip › jcm-4403372-supplementary.pdf]

Supplementary Materials

Real-World Safety and Herpes Zoster Outcomes After Recombinant Zoster Vaccination in Inflammatory Bowel Disease

Authors: Gian Mario Morrone, Sara Sandri, Marta Vernero, Angelo Armandi, Gian Paolo Caviglia, Davide Giuseppe Ribaldone

Correspondence: gianmario.morrone@unito.it

Contents

- Supplementary Table S1. Baseline characteristics and follow-up
- Supplementary Table S2. IBD therapy at the start of follow-up
- Supplementary Table S3. Time-dependent Cox proportional hazards models
- Abbreviations

Supplementary Table S1

Baseline characteristics and follow-up according to vaccination status.

Variable	Vaccinated (n = 69)	Not vaccinated (n = 45)	p-value
IBD duration, years	11 (3–20)	11 (3–18)	0.46
Age at follow-up start, years	47 (35–58)	45 (35–62)	0.78
Follow-up duration, months	16 (8–24)	23 (10–34)	0.02
Female sex	33 (47.8%)	18 (40.0%)	0.45
Male sex	36 (52.2%)	27 (60.0%)	—
Crohn's disease	44 (63.8%)	32 (71.1%)	0.54
Ulcerative colitis	21 (30.4%)	13 (28.9%)	—
IBD-U	4 (5.8%)	0 (0%)	—
Prior herpes zoster	14 (20.3%)	9 (20.0%)	1.00
Advanced therapy	42 (60.9%)	30 (66.7%)	0.56
Baseline HBI in CD	2 (2–3)	2 (2–3)	0.43
Baseline partial Mayo in UC/IBD-U	3 (2–3)	2 (2–2)	0.09

Note. Values are expressed as median (IQR) or n (%). Advanced therapy includes biologic agents and small molecules; azathioprine was classified as a conventional immunomodulator. IBD-U, inflammatory bowel disease-unclassified; HBI, Harvey–Bradshaw Index; IQR, interquartile range.

Supplementary Table S2

IBD therapy at the start of follow-up according to vaccination status.

Therapy	Vaccinated	Not vaccinated
Adalimumab	19 (27.5%)	11 (24.4%)
Azathioprine	1 (1.4%)	0 (0%)
Filgotinib	1 (1.4%)	0 (0%)
Infliximab	4 (5.8%)	9 (20.0%)
Risankizumab	2 (2.9%)	1 (2.2%)
Tofacitinib	1 (1.4%)	0 (0%)
Upadacitinib	3 (4.3%)	0 (0%)
Ustekinumab	7 (10.1%)	4 (8.9%)
Ustekinumab + vedolizumab	1 (1.4%)	0 (0%)
Vedolizumab	4 (5.8%)	5 (11.1%)
No advanced/conventional therapy	26 (37.7%)	15 (33.3%)
Any advanced therapy	42 (60.9%)	30 (66.7%)
Total	69 (100%)	45 (100%)

Note. Percentages are calculated using the column denominator. The row 'Any advanced therapy' is a summary category and should not be summed with the individual therapies.

Supplementary Table S3

Time-dependent Cox proportional hazards models for patient-reported herpes zoster.

Model and variable	HR (95% CI)	p-value
Model 1: Vaccination (time-varying)	2.29 (0.61–8.65)	0.22
Model 1: Prior herpes zoster	6.26 (1.80–21.76)	0.004
Model 2: Vaccination (time-varying)	2.51 (0.66–9.54)	0.18
Model 2: Advanced therapy	0.66 (0.20–2.17)	0.49

Note. Vaccination was modelled as a time-varying exposure beginning 14 days after the documented second dose. CI, confidence interval; HR, hazard ratio.

Abbreviations

Abbreviation	Definition
CI	Confidence interval
HBI	Harvey–Bradshaw Index
HR	Hazard ratio
IBD	Inflammatory bowel disease
IBD-U	Inflammatory bowel disease-unclassified
IQR	Interquartile range
RZV	Recombinant zoster vaccine
UC	Ulcerative colitis
